# How Clustered DNA Damage Can Change the Electronic Properties of ds-DNA—Differences between GAG, GA^OXO^G, and ^OXO^GA^OXO^G

**DOI:** 10.3390/biom13030517

**Published:** 2023-03-11

**Authors:** Boleslaw Karwowski

**Affiliations:** DNA Damage Laboratory of Food Science Department, Faculty of Pharmacy, Medical University of Lodz, ul. Muszynskiego 1, 90-151 Lodz, Poland; boleslaw.karwowski@umed.lodz.pl

**Keywords:** Base Excision Repair, 7,8-dihydro-8-oxo-2′-deoxyguanosine, electron hole and excess electron transfer, reactive oxygen species, electronic properties, DFT

## Abstract

Every 24 h, roughly 3 × 10^17^ incidences of DNA damage are generated in the human body as a result of intra- or extra-cellular factors. The structure of the formed lesions is identical to that formed during radio- or chemotherapy. Increases in the clustered DNA damage (CDL) level during anticancer treatment have been observed compared to those found in untreated normal tissues. 7,8-dihydro-8-oxo-2′-deoxyguanosine (^OXO^G) has been recognized as the most common lesion. In these studies, the influence of ^OXO^G, as an isolated (oligo-^O^G) or clustered DNA lesion (oligo-^O^G^O^G), on charge transfer has been analyzed in comparison to native oligo-G. DNA lesion repair depends on the damage recognition step, probably via charge transfer. Here the electronic properties of short ds-oligonucleotides were calculated and analyzed at the M062x/6-31++G** level of theory in a non-equilibrated and equilibrated solvent state. The rate constant of hole and electron transfer according to Marcus’ theory was also discussed. These studies elucidated that ^OXO^G constitutes the sink for migrated radical cations. However, in the case of oligo-^O^G^O^G containing a 5′-^OXO^GA^XOX^G-3′ sequence, the 3′-End ^OXO^G becomes predisposed to electron-hole accumulation contrary to the undamaged GAG fragment. Moreover, it was found that the 5′-End ^OXO^G present in an ^OXO^GA^OXO^G fragment adopts a higher adiabatic ionization potential than the 2′-deoxyguanosine of an undamaged analog if both ds-oligos are present in a cationic form. Because increases in CDL formation have been observed during radio- or chemotherapy, understanding their role in the above processes can be crucial for the efficiency and safety of medical cancer treatment.

## 1. Introduction

**Figure 1:**
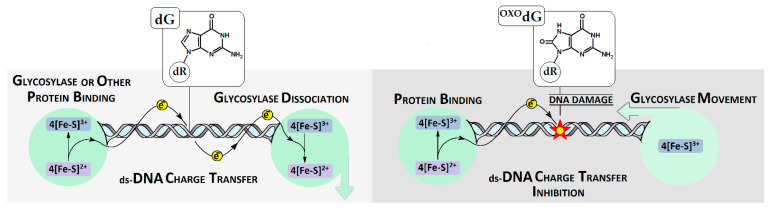
Schematic overview of [4Fe-4S] protein communication and DNA damage recognition by glycosylases via charge transfer mode. The unimpaired electron transfer through ds-DNA between two proteins (**right**) and electron transfer quenching (**left**) by DNA damage, which force the glycosylases to recruitment and action. dG: 2′-deoxyguanosine; ^OXO^dG: 7,8-dihydro-8-oxo-2′-deoxyguanosine; dR: deoxyribose.

The genetic information that is housed in the nucleus is continuously exposed to harmful extra- and intracellular factors such as UV or reactive oxygen, and nitric species on the metabolic product. Moreover, DNA damage can be the result of direct or indirect ionization radiation (gamma rays, X-rays, etc.). However, the formed lesions are structurally similar and undergo the same repair processes [1]. The richest nucleobase in terms of electrons is 2′-deoxyguanosine (dG), which makes it the most easily prone to oxidation. The loss of the electron by dG leads to radical cation formation, which can be converted by the reaction with a water molecule to ^OXO^dG (7,8-dihydro-8-oxo-2′-deoxyguanosine), or by proton abstraction to the dG neutral radical. On the other hand, dG can react with a hydroxyl radical (the most reactive species known to chemists), which also leads to ^OXO^G formation [2,3]. As has been reported, the 7,8-dihydro-2′-deoxyguanosine shows a lower ionization potential (IP) than dG, and thus can be perceived as a sink for the radical cation migrating through the double helix (electron hole) [4]. The systematic studies of Sugijama and Saito showed that as the guanine numbers increase, the ionization potential decreases: G > GG > GGG > GGGG [5]. These studies were complemented by Voityuk and Siebbeles, who calculated the IP for all base combinations in the trimer [6,7]. The lowest vertical IP (VIP) was assigned as follows: GGG, AGG, GGA, and AGA. These data suggest that the presence of such a sequence in a double helix determines the region of ^OXO^G appearance with a high probability. It is important to mention that a 7,8-dihydro-2′-deoxyguanosine triphosphate (^OXO^dGTP) from the cellular pool can be incorporated into the genome by polymerases, leading to increases in the level of ^OXO^dG [8]. This abundant lesion (200 per cell per hour) is effectively removed from oligonucleotides by bifunctional glycosylase OGG1, which initiates the Base Excision Repair System (BER). However, ^OXO^dG can form the non-canonical base pair with dA, which if unrepaired, can lead to GC→TA transversion. However, dA from this ^OXO^G::A base pair is removed by specific MutY glycosylases involved in the GO system [9]. Recently, Jacqueline Barton postulated that this specific enzyme, which contains [4Fe-4S] clusters, can recognize lesions by double helix electron scanning (Figure 1) [10]. This hypothesis effectively explains the efficiency of BER enzymes, which can scan the *Escherichia coli* genome within minutes. Even if the charge transfer through unmodified ds-DNA is recognized, the role of the different isolated and clustered lesions is still obscured. It should be mentioned here that charge transfer takes place between stacked base pairs, which constitute the central part of ds-DNA, while the sugar-phosphate backbone stabilizes the double helix geometry. The recent studies of Georgakilas et al. showed that the formation of the apurinic/apyrimidinic site in the cluster mode leads to significant double helix distortion, which can significantly influence the DNA damage recognition/repair process [11]. In this article, the influence of local multi-damage sites containing two ^OXO^dG on charge transfer and the electronic properties of short ds-DNAs were considered using the density functional theory (DFT). The obtained results are discussed in comparison with data obtained for unmodified analogs.

## 2. Materials and Methods

The initial structures of short ds-DNAs were built using the BioVia Discovery Studio v20.1.0.19295 software [12] and noted as follows: d[A_1_G_2_A_3_G_4_A_5_]*d[T_5_C_4_T_3_C_2_T_1_], d[A_1_G_2_A_3_^O^G_4_A_5_]*d[T_5_C_4_T_3_C_2_T_1_], and d[A_1_^O^G_2_A_3_^O^G_4_A_5_]*d[T_5_C_4_T_3_C_2_T_1_] as oligos: oligo-G, oligo-^O^G, and oligo-^O^G^O^G respectively.

The negative charges of the phosphate groups were neutralized by the addition of protons, and the other atoms were saturated by additional hydrogen atoms as necessary. The structure optimizations of *ds*-pentamers were performed using the QM/MM strategy. The structures of *ds*-oligo were divided into high-HL (nucleobases, M06-2X/D95**), and low-LL (sugar-phosphate backbone, M06-2X/sto-3G) levels of calculation using the ONIOM (Our own N-layered Integrated Molecular Orbital and Molecular Mechanics) method [13,14,15]. All calculations were performed in the condensed phase using Tomasi’s polarized continuum model (PCM) [16]. All energy calculations were performed in the aqueous phase according to the density functional theory (DFT) using the M062x functional with an augmented polarized valence double-ζ basis set 6-31+G** for complete oligonucleotides and 6-31++G** after sugar-phosphate backbone removal [17]. The sugar-phosphate backbone was removed from the obtained structures, leaving suitable base pair systems with subsequent atom saturation with the necessary hydrogen atoms. The spatial locations of the hydrogen atoms added for saturation were optimized at the M06-2X/D95** level of theory in the aqueous phase, with the position of all other atoms frozen. For all the optimized geometries, a charge and spin analysis was achieved using Hirshfeld methodology at the M06-2X/6-31++G** level of theory [18]. The electronic properties of molecules were calculated as described previously: for further details, please see references [18,19]. The characterization of the transition dipole moment of excited states and the single point calculation at the M06-2X/6-31++G** level of theory were performed using time-dependent DFT (TD-DFT) methodology [20]. The electron coupling was calculated using the generalized Mulliken–Hush methodology [21]. The solvent effect was looked at in two modes following a previously described methodology: the non-equilibrium (NE) and equilibrated (EQ) polarizable continuum model (PCM) [22]. The energy of molecules in non-equilibrated solvent mode was calculated using two-step processes according to the save-read procedures. The following energy notation was used, i.e., the *E*_geometry_^charge^ of the molecule (neutral form) is described as *E*_0_^0^, the vertical cation/anion as *E*_0_^+^/*E*_0_, the adiabatic cation/anion as *E*_+_^+^/^+^*E*_−_^−^, and the vertical neutral formed from cation/anion state as *E_0_*^+^/*E*_0_^−^. The difference, given in eV, between the mentioned energies corresponds to the suitable electronic states described as follows: VIP-NE = *E*_0_^+(NE)^–*E*_0_^0^ (vertical ionization potential in the NE state); VIP-EQ = *E*_0_^+(EQ)^–*E*_0_^0^ (vertical ionization potential in the EQ state); AIP = *E_+_*^+^–*E*_0_^0^ (adiabatic ionization potential); VEAE-NE = *E_+_*^+^–*E_+_*^0(NE)^ (vertical electron attachment energy in the NE state); VEAE-EQ = *E_+_*^+^–*E_+_*^0(EQ)^ (vertical electron attachment energy in the EQ state); VEA-NE = *E*_0_^−(NE)^–*E*_0_^0^ (vertical electron affinity in the NE state); VEA-EQ = *E*_0_^–(EQ)^–*E*_0_^0^ (vertical electron affinity in the EQ state); AEA = *E*_0_^0^–*E*_−_^−^ (adiabatic electron affinity); VEDE-NE = *E*_–_^–^–*E*_−_^0(NE)^ (vertical electron detachment energy in NE state); VEDE-EQ = *E*_–_^–^–*E*_−_^0(EQ)^ (vertical electron detachment energy in EQ state). All the above calculations were performed with the Gaussian G16 (version C.01) software package [23].

## 3. Results

Up to now, more than 70 types of DNA damage have been detected [1]. The most intensively investigated is ^OXO^dG. This lesion is removed from the genome by the specific glycosylase OGG1, the activity of which initiates a cascade of BER protein activity. On the other hand, ^OXO^dG can form a non-canonical base pair with 2′-deoxyadenosine (dA), which is removed by MutY [24]. The abundance of oxidized dG in the genome raises the question of how enzymes recognize DNA lesions in the plethora of unmodified nucleosides in an effective way. One of the explanations is the scanning of a double helix by charge transfer between two proteins at a distance of thousands of bases. (This mechanism was proposed by Barton [25].) In these studies, the structural, electronic, and charge transfer differences between native ds-DNA, ds-DNA with single ^OXO^dG, and ds-DNA containing two ^OXO^dG separated by single dA were taken into consideration.

### 3.1. The Structure Changes in the DNA Double Helix after Electron Loss or Extra Electron Adoption

For these studies, the following short ds-oligo were chosen: d[AGAGA]*d[TCTCT], d[AGA^O^GA]*d[TCTCT], and d[A^O^GA^O^GA]*d[TCTCT], denoted as oligo-G, oligo-^O^G, and oligo-^O^G^O^G, respectively. The appearance of the additional oxygen atom in position C8 of guanine did not significantly change the spatial structure of the double helix. This means that this function is directed outwards from the helix, leaving the hydrogen bond and base stacking integration almost unaffected. The calculated Root Mean Square Deviation (RMSD) of the overlapping neutral structures of the discussed DNAs showed only negligible differences at the level of 0.072 and 0.172 [Å^2^] for oligo-G versus oligo-^O^G and oligo-G versus oligo-^O^G^O^G, respectively. In terms of hole or electron migration, the most interesting structural question is how the double helix compensates for the excess charge. The double helix contains two structural subunits, i.e., the internal part constructed by complementary base pairs, which interact via π-stacking, and the external sugar-phosphate backbone, which interacts with the first solvation layer and compensates the inter-shape geometry changes. Given the above, a comparative structural analysis was performed. A geometries comparison, based on the RMSD, of a neutral molecule with a molecule in anion forms, revealed the following order of structural resistance towards the excess electron: oligo-^O^G^O^G > oligo-^O^G > oligo-G. This order of changes was observed independently of the base, sugar-phosphate, or whole structure comparison. It should be pointed out that the obtained RMSD adopted values in the range of 0.11 to 0.17 [Å^2^] (Table 1), which indicated that the appearance of an excess electron in the system, did not force a significant geometry fluctuation.

The situation becomes the opposite when a molecule such as ds-DNA loses an electron. In such a situation, the radical cation is formed and starts to migrate until settled in a suitable place [26,27]. The careful structural analysis revealed that the structural changes forced by the electron loss are almost three times higher compared to those of the radical anion, irrespective of whether “complete” or “partial” DNA geometry was taken into consideration (Table 1). Additionally, the oligo-^O^G containing a single ^OXO^G turned out to be the most resistant to electron loss. The geometry of the radical cation of oligo-G showed significant changes in comparison with the neutral form. Surprisingly, the RMSD value found for oligo-^O^G^O^G was moderately lower than that obtained for unmodified ds-DNA. However, in all the discussed cases, a higher structural fluctuation was assigned for the sugar-phosphate spine. These observations indicate that structural changes in the internal part of the double helix, which directly influence stacking and hydrogen bond interaction, are compensated by the high flexibility of the sugar-phosphate skeleton.

### 3.2. The Oligo-G, Oligo-^O^G, and Oligo-^O^G^O^G Electronic Properties

**Figure 2:**
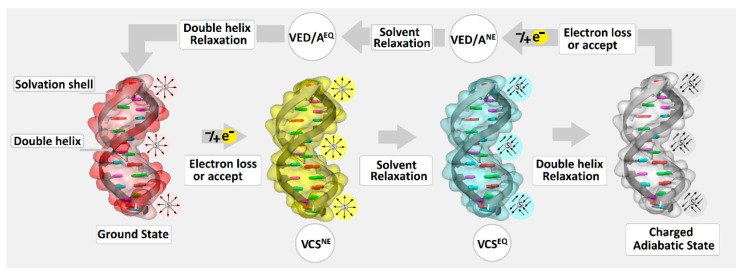
Schematic representation of electron hole or excess electron movement during charge transfer through a DNA double helix, and electronic parameters of this process in respect of the state of solvation environment. **VCS^NE^**—vertical charge state in non-equilibrated solvent mode, **VCS^EQ^**—vertical charge state in an equilibrated solvent state, **VED/A^NE^**—vertical state after electron detachment/attachment in non-equilibrated solvent mode, **VED/A^EQ^**—vertical state after electron detachment/attachment in equilibrated solvent mode.

The ds-oligo can be recognized as a nanowire in which charge migrates via a non-covalent interaction between stacked base pairs [28]. As shown by Barton et al., the charge (as a radical cation or anion) migrates through ds-oligonucleotide at a distance of hundreds of angstroms [29], by a hopping mechanism or over a few base pairs by a tunneling effect. The DNA damage appearing in the genome possesses the probability potential to upset the electron or hole migration through double-stranded nucleic acids. For example, the presence of ^OXO^dG, whose ionization potential value within canonical nucleotides is the lowest, in the double helix constituted the place of radical cation stabilization [30]. Therefore, DNA damage can influence charge migration by its electronic properties, such as ionization potential and electron affinity, without significant changes to the base pair stacking interaction. Interestingly, Gs separation by adenine (Ade) (GAG) leads to significant IP increases by 0.442 eV, while for the AGA, this value was found as 0.13 eV [6]. Therefore, from the charge transfer perspective, the presence of these two trimers in the ds-DNA structure (AGAGA) merits investigation. It should be pointed out that ds-oligo is surrendered by the first solvation shell, which stabilizes the double helix structure. Additionally, this water layer plays a significant role in ds-DNA/charge interaction and influences ionization potential and electron affinity (Figure 2) [31]. From the charge transfer perspective, it should be mentioned that the nucleus moves much more slowly than electrons (Franck–Condon rules). Therefore, the charge migration should be taken into consideration in light of the non-equilibrated solvent state. This solvent state (solvent–solute interaction) describes the initial molecular point of electron loss or adoption in the system. Moreover, the analysis of the vertical radical cation and anion of the non-equilibrated state of the short fragment of ds-DNAs reveals the following order of VIP^NE^: oligo-G > oligo-^O^G^O^G > oligo-^O^G, and (VEA^NE^): oligo-G ≈ oligo-^O^G > oligo-^O^G^O^G, respectively (Table 2).

As can be expected, a similar order was also found for the exclusively base pair system. The difference between both systems (ds-DNA and base pairs) in all cases was assigned at the level of ~0.2 eV. It indicates that in the discussed non-equilibrated solvent state, the nucleic bases are mainly responsible for the electronic properties of the double helix. Moreover, the solvent relaxation (solvent equilibrate (EQ) state) leads to the lowering of the vertical ionization potential and electron affinity values calculated for ds-DNA, by ~0.55/eV and ~0.60 eV, respectively. The same order of VIP^EQ^ was found for non-equilibrated states. Surprisingly, for VEA^EQ^ a higher value was found for oligo-G following the oligo-^O^G and oligo-^O^G^O^G in each case (Table 2). However, these differences were noted as negligible. After structure relaxation and adiabatic cation formation by ds-oligo, the following adiabatic ionization potentials in [eV] were assigned: 5.65/5.58 oligo-G; 5.39/5.38 oligo-^O^G; and 5.39/5.40 oligo-^O^G^O^G for ds-DNA and BP-system, respectively. As expected, the appearance of a single ^OXO^dG in the oligo structure caused ionization potential decreases by approximately ~0.2 eV of the ds-oligo under discussion. Surprisingly, the appearance of a second ^OXO^dG in ds-oligo did not lead to AIP descent. From the work of Sugijama and Saito it was expected [5], by analogy, that the additional ^OXO^dG in proximity to the other should lead to AIP descent of the ds-DNA fragment, but no such observation was noted (Table 2).

Conversely, the electron attachment by the system after structure relaxation leads to adiabatic radical anion formation. As shown in Table 2, in each case (i.e., oligo-G, oligo-^O^G, and oligo-^O^G^O^G), the adiabatic electron affinities were found at the same level, i.e., −2.0 eV and −1.9 eV (with and without a sugar-phosphate backbone). In light of the charge migration through the double helix, the end of the process can be described by vertical electron detachment (VEDE) or attachment energy (VEAE) in equilibrated and non-equilibrated solvent states. As expected, the VEAE^NE^/^EQ^ values calculated for oligo-G adopted the highest value (4.61/5.26 eV), which indicates that the electron attachment process is the most privileged within the discussed ds-oligos. Oligo-^O^G and oligo-^O^G^O^G have similar values, and no influence on the sugar-phosphate backbone was observed.

### 3.3. Ds-DNA Changes in Charge, Spin, and HOMO–LUMO Distribution

The electronic properties of the ds-oligo discussed above describe the outcome of several—in this case, five—nucleosides linked by the phosphate group in a DNA B-form. It should be mentioned here that the outer layer of the *ds*-oligos structure interacts continuously with the solvation layer [31]. Therefore, the initial step of charge migration, HOMO–LUMO (high occupied molecular orbitals–low unoccupied molecular orbitals), as well as charge and spin distribution should be discussed with reference to the non-equilibrated/equilibrated solvation modes [16]. This approach can elucidate the subtle influence of DNA damage on charge transfer through the double helix. For these studies, the stacked base pairs through which cation and anion radicals have been taken into consideration pass. It is commonly accepted that the sugar-phosphate backbone does not play a significant role in charge migration [32]. The analysis of Hirshfeld charge distribution, calculated at the M062x/6-31++G** level of theory, showed that at the initial point of hole formation, charge and spin are dispersed over three bases. This is in good agreement with previous data [31]. An analysis of the vertical cation radical state of oligo-G in a non-equilibrated solvent state showed that charge/spin is distributed as follows (%): G_2_C_4_ (39/39), A_3_T_3_ (8/9), and G_4_C_2_ (47/48). As can be expected, the appearance of ^OXO^G in the oligo-^O^G structure leads to charge and spin accumulation of 80/89 (%) on the ^OXO^G_4_C_2_ base pair. These results correspond well with the fact that ^OXO^G showed a lower ionization potential than guanine (discussed below) [33]. Surprisingly, the second ^OXO^G appearing in the ds-DNA structure, i.e., oligo-^O^G^O^G, led to a similar result as that noted for unmodified oligo-G. The charge and spin settled mainly on ^OXO^G_2_C_4_ (48/43(%)), ^OXO^G_4_C_2_ (39/40(%)), and, in a small amount, on A_3_T_3_ (9/10(%)). After solvent and solute relaxation, the charge and spin in the vertical (equilibrated state) and adiabatic form of radical cation was found exclusively on G_2_C_4_ for oligo-G and on ^OXO^G_4_C_2_ of oligo-^O^G, which was expected. The G located at the 5′-End on multi Gs sequences (GG, GGG, etc.) is much more prone to oxidization than others guanines. However, in the system where two ^OXO^G are present in both the discussed solvent states, the charge and spin were dispersed over the ^OXO^G_2_C_4_ and ^OXO^G_4_C_2_ moieties of oligo-^O^G^O^G. It should be pointed out that the solvent relaxation and conversion between vertical cation radical forms from a non-equilibrated to equilibrated state, force scant changes in charge/spin distribution, as shown in Table S1. After oligo-^O^G^O^G’ structure relaxation towards the adiabatic form, the charge and spin shifted to the ^OXO^G_4_C_2_ base pair, leaving approximately 13/10 (%) on the ^OXO^G_2_C_4_ moiety. These observations show that the proximity of two 8,7-dihydro-8-oxo-2′-deoxyguanosines in the oligo structure should lead to the “island of hole stabilization” appearing. Moreover, the ^OXO^G located on the 3′-End site of ds-oligo containing the ^OXO^GA^OXO^G part is privileged for further oxidizing, which is contrary to the observation obtained previously for native ds-oligo containing GAG sequences. 

**Figure 3:**
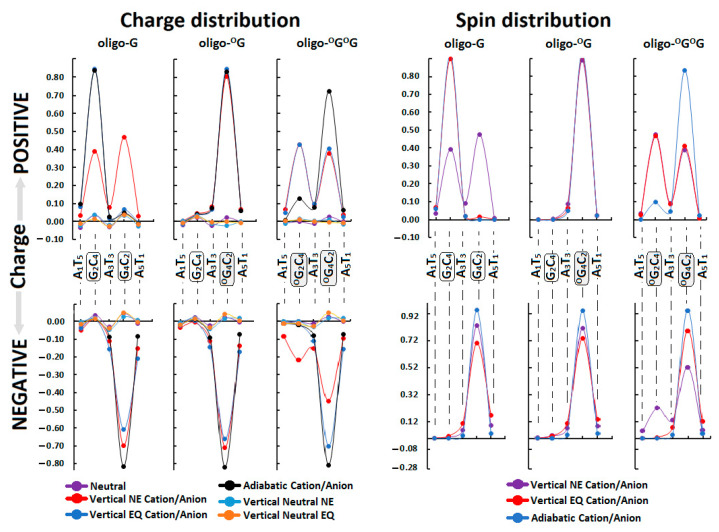
Spin and charge distribution in ds-DNA: native oligo-G, containing ^OXO^G as an isolated lesion oligo-^O^G and containing two ^OXO^G as a clustered DNA lesion oligo-^O^G^O^G in their neural, vertical cation/anion, adiabatic cation/anion, and vertical neutral forms, calculated at the M062x/6-31++G** level of theory in the aqueous phase. Solvent EQ (equilibrated) and NE (non-equilibrated) states were taken into consideration. ^OXO^G is indicated as ^O^G to simplify the notation. The row data have been given in Table S2.

The situation became different when an extra electron was introduced into the discussed systems. The negative charge and spin were mainly located on the X_4_C_2_ base pair (X_4_ = G or ^OXO^G) in adiabatic stages of all the discussed ds-oligos as follows (%): 82/94 (oligo-G), 82/94 (oligo-^O^G), and 81/94 (oligo-^O^G^O^G). It should be pointed out here that no significant influence of solvent relaxation on the charge and spin distribution was observed. Only for oligo-^O^G^O^G was a negative charge and spin dispersion over ^O^G_2_C_4_ (22/22%) and ^O^G_4_C_2_ (45/52%) observed in the non-equilibrated solvent state (Figure 3, Table S1).

**Figure 4:**
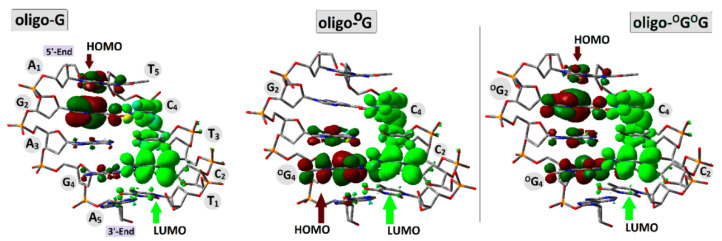
Graphical representation of the molecular orbital distributions (HOMO, LUMO), calculated at the M062x/6-31++G** level of theory in the condensed phase (equilibrated mode) of oligo-G, oligo-^O^G, and oligo-^O^G^O^G. Dark green/brown indicated HOMO distribution while Light green indicated LUMO.

The analysis of the HOMO–LUMO distribution of neutral oligos in their ground states supports the previously mentioned results well. To complement the discussion, the integral structures of ds-DNAs were taken into consideration, i.e., those containing a sugar-phosphate backbone and base pairs. In the neutral state (adiabatic), HOMO is located/distributed on the following base pairs: G_2_C_4_ of oligo-G, ^OXO^G_4_C_2_ of oligo-^O^G, and ^OXO^G_2_C_4_ and ^OXO^G_4_C_2_ of oligo-^O^G^O^G (Figure 4). The above corresponds well with the charge and spin distribution of the adiabatic radical cation state (Figure 3). Moreover, the similar distribution of LUMO indicated that the negative charge and spin of the adiabatic radical anion should show a similar character, as has been discussed previously. Therefore, the above observations are in good agreement with Kohn–Sham’s theory [34].

### 3.4. A Comparison of the Electronic Properties of Individual Base Pairs within Oligo-G, Oligo-^O^G, and Oligo-^O^G^O^G

As shown above, the high occupied molecular orbital is mainly located in all the discussed ds-oligos (oligo-G, oligo-^O^G, and oligo-^O^G^O^G) on the purine strand, while LUMO is on the pyrimidines one. This corresponds well with radical anion and cation distribution. These observations are in good agreement with Sevilla’s previous studies, which showed that during oligonucleotide γ-radiation, the following amounts of suitable radicals are formed: 35% G^●+^, 5% A^●+^, and about 45% of T^●−^ and C^●−^ [35]. The electronic properties of nucleic bases, nucleosides, and their pairs have been intensively studied during the last decades using theoretical and experimental methods. However, data numbers concerning the DNA damage are low, and most of them discussed mainly ^OXO^G as an isolated lesion [31,36]. Due to system complications, the vertical ionization potential of nucleobase pairs was willingly analyzed. Rotshilberg utilized VIP to predict charge transfer [36,37]. As shown above, the appearance of the ^OXO^G in the double helix structure changes the electronic properties depending on the DNA damage type: isolated or clustered. Moreover, following Sevilla, it can be concluded that the equilibrated or non-equilibrated solvent state plays a significant role in ds-DNA electronic properties’ determination [31]. The situation becomes more complicated when the isolated form of double helix base/nucleoside pairs are taken into consideration. As shown in Figure 2, the non-equilibrated solvent mode can be used when the whole molecule is plunged into the solvent shell as ds-DNA (solvation layer). It is assumed that each AT and GC pair, in the B-form of ds-oligo, contained 44 and 27 H_2_O molecules, respectively [38]. However, no water molecules are present in the space between the stacked bases, which complicated the usefulness of the non-equilibrated mode for this study. Therefore, for further calculations, the equilibrated mode was used as a good approximation for solitary base pairs extracted from the ds-DNA structure.

The obtained results reveal the difference in the vertical ionization potential (VIP) of single base pairs depending on the investigated ds-oligo. The lowest VIP was found for G_2_C_4_ and G_4_C_2_ of oligo-G. Surprisingly, these values were equal for both of them, i.e., 6.13 eV, which indicates that the charge is distributed over the distance of three base pairs. The situation is similar, as expected, in the case of oligo-^O^G^O^G; however, a slightly lower VIP was noted for ^OXO^G_4_C_2_ (5.91 eV) than for ^OXO^G_2_C_4_ (5.93 eV). According to the previous studies, a significant decrease in VIP was observed exclusively for ^OXO^G_4_C_2_ of oligo-^O^G, which confirms that the solitary ^OXO^dG appearing in an oligo structure can play the role of a radical cation sink [39]. After electron loss and double helix structure reorganizations (relaxation), the lowest calculated adiabatic ionization potentials (AIP) were found for G_2_C_4_ of oligo-G, ^OXO^G_4_C_2_ of oligo-^O^G, and ^OXO^G_4_C_2_ of oligo-^O^G^O^G as follows: 5.83, 5.56, and 5.56 (eV), respectively. These results elucidated the difference in electronic properties between oligo-G and oligo-^O^G^O^G where the dGs or ^OXO^dG exist in some kind of resonance. This is in good agreement with the above-mentioned charge, spin, and HOMO localization. This observation indicates that in clustered DNA damage, the charge distribution and its transfer are different than was noted for unmodified ds-oligo. Following the studies of Saito and Sugiyama, the hole is mainly located on the 5′-End G of the GG, GGG sequence [5,40]. A similar result was obtained here for oligo-G, while for oligo-^O^G^O^G, the situation was the opposite, i.e., the hole was mainly accumulated on the 3′-End ^O^G. These observations are important for further predicting the “cation radical hot point” of the genome as the most fragile for ionization during radiotherapy, for example. A comparison of the AIPs of the base pairs of oligo-G and oligo-^O^G^O^G shows that the G_2_C_4_ base pair has a lower value than ^OXO^G_2_C_4_ in the adiabatic radical cation forms of the discussed oligos: 5.83 vs. 5.93 (eV). It is important to mention here that oligo-G and oligo-^O^G^O^G contained a repeated sequence as follows: AGA and A^O^GA. Voityuk and Gurea discussed the ionization potential of the above trimers as isolated molecules in the context of all the available trimers [6,7]. Here, it has been found that in the case of complex ds-DNA trimers, A_1_G_2_A_3_ and A_3_G_4_A_5_ as well as A_1_^O^G_2_A_3_ and A_1_^O^G_2_A_3_ are not equal. These results indicate that CDL can play a significant role in charge transfer and, subsequently, in DNA damage recognition, changing the “rules of the game”. Moreover, the lower absolute values of vertical electron attachment energies (VEAE) were found for base pairs for which lower AIPs were noted. This directly indicates the point at which significant ds-oligo structural changes take place, which was confirmed by higher *λ* values (Table 3). As shown in Table 3, the changes between VIP and AIP were denoted only for G_2_C_4_(oligo-G) and ^OXO^G_4_C_2_(oligo-^O^G, -^O^G^O^G). For the other base pairs of all the ds-oligos, the VIP, AIP, and VEAE were assigned as almost equal (the differences can be neglected). These results indicate that hole transfer can be performed without structural changes in ds-oligo until the destination point has been reached, which is in good agreement with the Franck–Condon principle [41]. The appearance of the additional electron in the double helix leads to vertical anion radical formation with subsequent conversion to its adiabatic forms. The assignment of vertical and adiabatic electron affinity energy (VEA, AEA) of the base pairs isolated from ds-oligo elucidate that in all cases, the extra electron is mainly settled on G_4_C_2_ and G_4_C_2_ of oligo-G and oligo-^O^G, oligo-^O^G^O^G. This is well supported by charge and spin distribution in the complete structure of ds-DNA. Moreover, in all cases, the vertical electron detachment energy (VEDE) adopts for these base pairs a higher value (~2.5 eV), which is typical for a point of radical accumulation in its adiabatic form. Surprisingly, in all the discussed cases, irrespective of the oligo, the VEA and AEA of G_4_C_2_ and ^OXO^G_4_C_2_ adopt almost the same values, namely −1.53 and −1.97 eV, respectively. This indicates that if glycosylases use the electron transfer for double helix scanning towards DNA damage localization, the ^OXO^dG radical cation can play a significant role as an electron trap. The calculations of the VAE, AEA, and VEDE parameters for the other base pairs isolated from oligo-G, oligo-^O^G, and oligo-^O^G^O^G had the same value in (eV) with negligible fluctuations, as shown in Table 3. The above observation indicates that electron transfer takes place without nucleus movement.

### 3.5. The Differences between Oligo-G, Oligo-^O^G, and Oligo-^O^G^O^G during Charge Migration through ds-DNA

**Figure 5:**
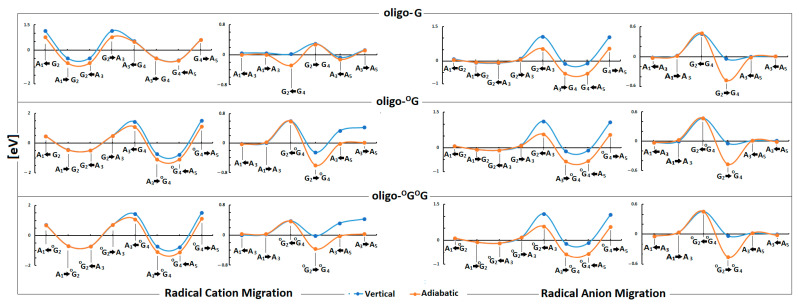
The energy barrier profile of the charge migration process through the native DNA double helix containing isolated and clustered lesions in vertical (blue line and spot) and adiabatic (orange line and spot) modes. The numeric data are given in Table S4.

The stacked base pairs in the double helix resulting from π-π interaction allow charge migration through DNA as a nanowire. As mentioned, the radical cation can travel a distance of over 200 Å, at least, from the place of one-electron oxidation [42]. These processes can be discussed in three categories: single-step tunneling, random-walk multistep, and polaron-like hopping [43]. The long-distance charge migration between G:::C pairs via incoherent mechanisms is strongly dependent on the bridge nature, usually A::T pairs. On the other hand, the tunneling path can be observed at a distance of only a few base pairs. However, irrespective of the charge transfer mechanism, the base pair’s mutual position, spatial geometry, and individual electronic properties are crucial. Therefore, it can be expected that DNA lesions can influence electron or hole migration through a double helix. It has been shown that the ^OXO^dG in ds-oligo is constituted as a stopping point for further charge migration [39]. Therefore, DNA damage can influence the charge migration not only by its disparate electronic properties but also by its numbers and distribution in the double helix turns. Surprisingly, as shown above, the 7,8-dihydro-8-oxo-2′deoxyguanosine did not change the ds-oligo spatial structure significantly—even in the clustered damage mode, i.e., oligo-^O^G^O^G. On the other hand, G’s separation by adenine in GAG leads to significant IP increases by 0.442 eV in comparison to GGG, while for the AGA this value was found to be 0.13 Ev [6]. Therefore, from the charge transfer perspective, the presence of these two trimers in the ds-DNA structure (native and with variable ^OXO^G numbers) is worth investigating. Based on molecular orbital distribution and inspection of the ionization potential and electron affinity of all the base pairs, it can be expected that the hole transfer mechanism in the discussed ds-DNAs should be different, while the electron migration should be almost identical. However, the different HOMO localization (in the neutral stage) and spin distribution in the vertical cation state suggest the significant role of the ^OXO^G in these processes (Figure 3 and Figure 4). The energy barriers of charge migration can be estimated in the vertical and adiabatic modes. In these studies, the investigated pentamers were divided into three trimers of oligo-G (X-G) and oligo-^O^G(X-^OXO^G): A_1_G_2_A_3_, G_2_A_3_X_4_, A_3_X_4_A_5,_ or A_1_X_2_A_3_, X_2_A_3_X_4_, A_3_X_4_A_5_ of oligo-^O^G^O^G. Accordingly, the barriers of radical cation/anion transfer can be assigned as follows (notification has been simplified to the nucleobases of the purine strand) for an iterative single-step super exchange process: A_1_^+^→X_2_^+^; A_1_^+^←X_2_^+^; X_2_^+^→A_3_^+^; G_2_^+^←A_3_^+^; A_3_^+^→X_4_^+^; A_3_^+^←X_4_^+^; X_4_^+^→A_5_^+^; X_4_^+^←A_5_^+^ and for hole/electron hopping A_1_^+^→A_3_^+^; A_1_^+^←A_3_^+^; X_2_^+^→X_4_^+^; X_2_^+^←X_4_^+^; A_3_^+^→A_5_^+^; A_3_^+^←A_5_^+^, depending on the ds-oligo X = G or X = ^OXO^G (Figure 5). Following the nature of the charge migration process, the energies of the donor and acceptor were described as the sum of the energies of suitable base pairs, extracted from an adequate pentamer, for example, the energy of donor A_1_(*E*_+_^+^), X_2_(*E*_0_^0^), A_3_(*E*_0_^0^) and acceptor A_1_(*E*_0_^0^), X_2_(*E*_+_^0^), A_3_(*E*_+_^+^). The energy “barrier” was described as the sum of A_1_(*E*_+_^0^), X2(*E*_0_^+^), A_3_(*E*_0_^0^) energies in the vertical mode or A_1_(*E*_+_^0^), X_2_(*E*_+_^+^), A_3_(*E*_0_^0^) in the adiabatic mode, as shown in Figure 5 and Table S2 and described previously [19].

The analysis of the ionization potential of guanine involved in different triplets, denoted as 5′-XGY, showed that the G located at the 5′-End adopts a lower IP value [44]. Additionally, an inspection of all the possible trimers indicated that the ability of the central base pair to lose an electron is strongly affected by the nature of the 3′-End base pair [45,46]. In a more complex system (oligo-G), which contained repeated trimers AGA in the vertical mode of charge single-step tunneling, both Gs (G_2_ and G_4_) constituted equal sinks (Δ*G* = −0.47 eV) of radical cation, separated by barriers Δ*G* = −1.14 eV and Δ*G* = −0.54 eV for G_2_→A_3_ and A_3_←G_4_, respectively. The details are presented below in Figure 5 and Table S2. After nucleus relaxation, the preferences of the hole settling shifted to the 5′G, i.e., G_2_(Δ*G* = −0.77 eV). Surprisingly, the process conversion from the vertical to the adiabatic mode of charge migration did not influence the Δ*G* of the unit step between the base pairs located on the 3′-End. The opposite was found for the G_2_C_4_ base pair located on the 5′-End purine strand side. A similar scheme was denoted for the hole hopping process between the base pairs located on the 5′ and 3′-End of adequate trimers. However, depending on whether the Δ*G* calculation mode was vertical or adiabatic, the following values were found: 0.02 eV or −0.28 eV, respectively, for radical cation transfer between G_2_ and G_4_ base pairs over the A_4_ bridge. Moreover, the obtained Δ*G* values for the other hole hopping steps were close to 0 eV in the vertical mode of calculation and slightly changed in the adiabatic mode for the A_3_→A_5_ (0.12 eV) and A_3_←A_5_ (−0.12 eV) steps. These results confirm that the positive charge migration through the native unmodified double helix can be extremely fast until the hole settles down on its preferred 5′-End guanine. The situation becomes different when a single ^OXO^G appears in ds-DNA as oligo-^O^G. Firstly, as expected, the direction of hole migration is privileged towards ^OXO^G_4_, and the lower Δ*G* values were denoted for (G_4_←A_4_) and (A_3_G_4_) as follows (vertical/adiabatic mode): −0.74/−1.09 (eV) −0.77/−1.09 (eV). A significant barrier for the charge migration in the opposed direction was assigned, 1.45/1.09 (eV) for G_4_→A_4_ transfer and 1.53/1.12 (eV) for A_3_←G_4_. For base pairs located on the 3′-End of oligo-^O^G, the analysis of the hole hopping mechanism elucidated a notable difference between the energy barrier calculated in the vertical and adiabatic modes. The situation is the opposite in the case of native oligo-G, where it was negligible, as shown in Figure 5 and Table S2. The above indicates that the radical cation easily reaches the ^OXO^G_4_ position and, due to high barriers, cannot escape from the energetic depression. The barrier profiles of hole migration after the conversion of both guanines to ^OXO^Gs, as in oligo-^O^G^O^G, by analogy, should be the same as those found for oligo-G. Surprisingly, though, a lower Δ*G* was obtained for A_3_→^OXO^G_4_ and ^OXO^G_4_←A_5_ (Figure 5 and Table S2). The above indicates that ^OXO^G_4_ located on the 3′-End of the ^OXO^G_2_A_3_^OXO^G_4_ is the radical cation point of destination instead of the 5′-End, which is commonly accepted for native G_2_A_3_G_4_. The hole-hopping mechanism had a similar energetic pattern as that found for oligo-^O^G. Moreover, after adiabatic radical cation formation on the ^O^G_4_C_2_ base pair, the Δ*G* of hole transfer from A_3_←A_5_ and A_3_→A_5_ as well as A_1_←A_3_ and A_1_→A_3_ adopts a value close to 0 (eV), which indicates that in this mode, the charge transfer becomes almost unaffected. The situation changes when the electron transfer was taken into consideration. The profile of the energy barrier was similar in each discussed case as shown in Figure 5. For the single-step mode, the lower energetic barrier (Δ*G*) was denoted for A_3_→^OXO^G_4_ and ^OXO^G_4_←A_5_ (oligo-^O^G^O^G) at a level of −0.1 and −0.50 (eV) for the vertical and adiabatic modes. The analysis of the hopping mechanism found that the electron transfer was privileged for the ^OXO^G_2_→^OXO^G_4_ transfer in each case (Figure 5, Table S2). Surprisingly, the barrier of electron hopping between adenines was assigned in the range of −0.04 to 0.01 (eV), irrespective of whether the base pairs were positioned on 3′ or 5′-Ends, and the ds-oligos were analyzed. The above results indicate that ds-oligo scanning by glycosylases, which utilizes electron transfer, does not depend on the mutual position of the protein and DNA lesion. These results are in good agreement with the previous data which show that electron transfer can occur between adenines almost without being affected [45]. Surprisingly, based on these results, it can be postulated that some lesions can be omitted by glycosylases since the electronic properties of ^OXO^G strongly depend on their neighborhood if it belongs to a repeated sequence, as shown in Figure 5.

### 3.6. The Rate Constant of Electron Hole and Excess Electron Transfer through Canonical and Lesioned ds-DNA

As shown, a charge transfer through a double-stranded oligonucleotide can occur in its oxidative or reduced state. This process was introduced and developed by Marcus [47,48]. According to this theory, the charge transfer depends on several factors: the rate constant (*k*_HT_), driving force Δ*G*, reorganization (*λ*)/activation (*E_a_*), and electron coupling (*V_da_*) energies. The rate constant links all the above parameters together, as shown in Equation (1), while the activation energy (*E_a_*) is described by Equation (2) (*k_b_* is the Boltzmann constant, *h*—is the Planck constant, and *T*—the temperature in (K)).

(1) $$k_{\mathrm{HT}}=\frac{4\pi {\left|V_{12}\right|}^{2}}{h}\cdot \sqrt{\frac{1}{4\pi \lambda k_{b}T}}\cdot \exp\left(\frac{-{\left(\Delta G+\lambda \right)}^{2}}{4\pi k_{b}T}\right)$$

(2) $$E_{a}=\frac{\lambda }{4}\cdot {\left(1+\frac{\Delta G}{\lambda }\right)}^{2}$$

(3) $$V_{12}=\frac{\Delta E_{12}\left|\mu_{12}\right|}{{\left(\mu_{1}-\mu_{2}\right)}^{2}+4\mu_{12}^{2}}$$

As shown by Equation (1), the charge transfer was influenced by electron coupling. These parameters (*V*_12_) are calculated according to the generalized Mulliken–Hush method (GMH) [21]. As described by Equation (3), *V*_12_ depends on the acceptor–donor diabatic transition dipole moment and vertical excitation energy of the radical cation/anion Δ*E*_12_ [21]. (In Equation (3), *μ*_1_–*μ*_2_ is the difference between the ground and first excited dipole moment, *μ*_12_—transition dipole moment.) For the charge transfer, one of the most important parameters is the reorganization energy (*λ*). The hole or electron passes through vertical states of the donor and acceptor associated with the rearrangement geometries of the solute in the polarization environment [49].

#### 3.6.1. The Driving Force

From the definition, the free energy difference between the initial and final state’s process is known as the “driving force” (Δ*G*), which is the difference in the redox potential of the investigated system [50]. For the canonical oligo-G, the highest Δ*G* values were found to be equal for the radical cation migration from A_1_T_5_ to G_2_C_4_ and from A_3_T_3_ to G_2_C_4_, i.e., 0.77 (eV) (Table 4). As expected, the hole hopping Δ*G* value noted for [G_4_C_2_]→[G_2_C_4_] was −0.28 (eV). The situation is the opposite in the cases of oligo-^O^G and oligo-^O^G^O^G, and the privilege value of Δ*G* calculated for A_3_T_3_→^O^G_4_C_4_ and G_4_C_4_←A_5_T_5_ was as follows: 1.09 and 1.12 (eV), respectively. Moreover, the hole-hopping driving force was privileged for G_2_C_4_→^O^G_4_C_2_ and ^O^G_2_C_4_→^O^G_4_C_2_ for oligo-^O^G (0.60 eV) and oligo-^O^G^O^G (0.38 eV). As discussed above, the equal highest Δ*G* values of electron transfer and electron hopping were found for oligo-G, oligo-^O^G, and oligo-^O^G^O^G, as follows in (eV): A_3_T_3_→^O^G_4_C_4_ (0.57), ^O^G_4_C_4_←A_5_T_5_ (0.56), G_2_C_2_→^O^G_4_C_4_ (0.47) (Table 4).

#### 3.6.2. Reorganization Energy

The charge migration forced geometry changes to the suitable base pairs donor (d) and acceptor (a), accompanied by nuclear reorganization energy (*λ*). The *λ* describes the energy changes in the adjacent structures, which occur during the hole or electron migration process (Table 4). The above-discussed parameter as well as activation energy have been calculated for the base pair’s structure isolated from the optimized ds-oligo, which can be different than that founded in the ideal model. It has been effectively shown previously for the AT cross-link studies [51].

For the radical cation transfer, the highest values of reorganization energy in (eV) were found as follows: oligo-G: A_1_T_5_→G_2_C_4_ (0.3) and G_2_C_4_←A_3_T_3_ (0.3), oligo-^O^G: A_3_T_3_→G_4_C_2_ (0.35) and G_2_C_4_←A_5_T_5_ (0.34), oligo-^O^G^O^G: A_3_T_3_→G_4_C_2_ (0.35) and G_2_C_4_←A_5_T_5_ (0.35) (Table 4). Additionally, the *λ* value assigned in (eV) for the G_2_C_4_←G_4_C_2_(0.29) hole transfer in oligo-G was lower than that found for G_2_C_4_→^O^G_4_C_2_ of oligo-^O^G and ^O^G_2_C_4_→^O^G_4_C_2_ of oligo-^O^G^O^G (0.35). These results elucidate the different electron-hole migrations within the double helix dependent on lesion distribution. The situation becomes simpler and more coincident when the electron transfer is considered. For all investigated ds-oligos, the same values were found for the A_3_T_3_→G_4_C_4_, G_4_C_4_←A_5_T_1_, and G_2_C_4_→G_4_C_2_ systems, as in Table 4. Moreover, the lack of significant changes in the nuclear reorganization energy (λs was found at the level of 0 eV) suggests that the hole transfer through the double helix almost passes undisturbed until it reaches a suitable destination, i.e., a G_4_C_2_ or ^OXO^G_4_C_2_ base pair. This is in good agreement with previous experimental results, which suggest that radical cation or excess electron transfer in the double helix can take place in almost distance-independent ways [52].

At this point, it is important to mention that even a low dose of radiation at the level of 1 Gy can induce clustered lesions [52,53]; hence, it can be expected that tandem lesions such as cdPus can be induced too. CdPus are produced in anaerobic conditions and a hypoxia milieu, which is common for cancer cells, and favors the reaction of carbon-centred or peroxyl nucleotide radicals with the adjacent DNA part [54]. In medical treatment, the most frequent radiodiagnostic techniques use X-rays and gamma radiation, which are classified as Low-LET (Linear Energy Transfer) factors. For example, 131I (a gamma and beta emitter) is commonly used for thyroid therapy and diagnosis [55]. On the other hand, medical centers based on High-LET treatment are becoming of high interest in nuclear medicine–radiotherapy. Following Asaithamby, it can be concluded that High-LET has induced numerous incidents of chromosomal rearrangement [56]. As of 2020, almost 100 such centers exist in 30 countries, with 200,000 patients treated in the USA alone [57]. Therefore, it is important to know how the repair proteins of primary and pathologic/cancer cells recognise the local multiple-damaged sites (LMDS) composed of different kinds of DNA lesions and how LMDS attract repair proteins. For example, a DNA double-strand break (DSB) or an inter- or intrastrand cross-link are repaired by homologous recombination (HR) and non-homologous end-joining (NHEJ) [58]. The above-mentioned repair mechanisms are already well recognised in the case of isolated lesions, while the recognition and repair process of clustered lesions still requires further advanced studies [59].

#### 3.6.3. Activation Energy

According to the Marcus theory of charge transfer, the activation energy *E_a_* (Equation (2)) can be expressed as a function of the two above-discussed parameters, i.e., *λ* and Δ*G* [60]. As presented in Table 4, the *E_a_* obtained at the level of M062x/6-31++G** in the condensed phase for the hole transfer process revealed the highest value (in eV) for A_3_T_3_→G_4_C_4_ (2.05) and G_4_C_4_←A_5_T_5_ (7.69) for oligo-G. The above suggests that hole transfer on the 3′-End of oligo-G (purine line) should be hindered and compensated by hole hopping between G_4_C_2_→G_2_C_4_ (*E_a_* = 0 was found). The ^OXO^G appearing in the oligo-^O^G reversed the above trend, with significant *E_a_* increases found for A_1_T_1_→G_2_C_2_ (11.08 eV) and G_2_C_2_←A_3_T_3_ (8.29 eV), while for G_2_C_2_→^OXO^G_4_C_4_ and A_1_T_1_←A_3_T_3_ they were, as previously, close to zero eV. The above observation suggests that radical cation transfer on the purine strand 3′-End of ds-oligo by a single-step tunneling process is unprivileged as opposed to electron hole hopping between guanine and 7,8-dihydro-8-oxo-2′-deoxyguanosine. The conversion of both guanines into ^OXO^G (oligo-^O^G^O^G) resulted in *E_a_* increases of up to 76.06 eV for G_2_C_2_←A_3_T_3_ with subsequent activation energy decreases for A_1_T_1_→G_2_C_2_ (−2.2 × 10^3^ eV). The results of *E_a_* calculations were as follows: 2 × 10^−4^ and 3.6 × 10^−4^ eV for A_1_T_1_←A_3_T_3_ and ^OXO^G_2_C_2_→^OXO^G_4_C_4_, respectively. This elucidated that the charge migration between adenines or guanines (or analogs) separated by an additional base pair can occur [61]. It should be pointed out here that *E_a_* adopted a negative value for A_1_T_1_→^OXO^G_2_C_2_ and A_3_T_3_←A_5_T_5_ transfer, which indicates that the discussed process should be highly privileged.

The analysis of the energy activation of a negative charge migration through stacked base pairs within oligo-G revealed a value at the same level, close to 0 eV, for all the discussed transfers (Table 4). A lower *E_a_* was found for G_2_C_2_→G_4_C_4_ (1.2 × 10^−4^ eV), while for the A_1_T_1_→G_2_C_2_, G_2_C_2_←A_3_T_3_, and A_3_T_5_←A_5_T_5_, the calculated activation energy adopted a negative value as for a strongly exothermic reaction. The above has been justified previously by Barton’s study, which proposed that an electron ejected from the [4Fe-4S] cluster (glycosylases cofactor) can travel through native ds-DNA at a distance of a thousand base pairs [62]. Similar results of the discussed process were found for oligo-^O^G and oligo-^O^G^O^G. However, significantly higher activation energy values in (eV), in comparison with native ds-oligo, were denoted for A_1_T_1_→G_2_C_2_ (0.21) of oligo-^O^G and A_1_T_1_→^OXO^G_2_C_2_ (0.75), and ^OXO^G_2_C_2_←A_3_T_3_ (0.60) of oligo-^O^G^O^G. The above indicates that electron transfer through the double helix via π-π base pair interaction can be slowed down when ^OXO^G appears within the system and can increase with its numbers. The above are in good agreement with the hypothesis that DNA damage can be recognized via electron transfer between two proteins containing [4Fe-4S] clusters [52].

#### 3.6.4. The Charge Transfer Rate Constant

All the parameters of charge transfer discussed above have been taken together in Equation (1). As shown, *k*_HT_ (the charge transfer rate constant) is strongly dependent on *E_a_*, Δ*G*, and *V*_12_ (electronic coupling matrix element) [60]. According to the fact that *k*_HT_ is strongly related to the spatial geometry changes in the donor and acceptor, the small influence of the ^OXO^G on charge transfer in the shape of a double helix can be expected in comparison to native ds-oligo (Table 4). The highest rate constants in (s^−1^) were found for A_1_T_1_→G_2_C_2_ and G_2_C_2_←A_3_T_3_ of unmodified oligo-G: 2.2 × 10^12^ and 1.5 × 10^12^, respectively. The appearance of ^OXO^G slows down the *k*_HT_ of single-step tunneling as follows: A_3_T_3_→^OXO^G_4_C_4_ (1.0 × 10^9^, 1.26 × 10^9^), ^OXO^G_4_C_4_←A_5_T_5_ (2.3 × 10^8^, 5.15 × 10^7^) for both oligo-^O^G and oligo-^O^G^O^G, respectively. Surprisingly, it was found that the *k*_HT_ of A_3_T_3_→^O^G_2_C_2_ transfer is close to 0 (s^−1^), making the hole migration almost impossible. Such a charge transfer can be compensated by the hole hopping mode between guanines or adenine moieties exclusively and the calculated rate constant adopted values in the range of 10^10^ to 10^14^ (s^−1^). However, the transfer between adenines A_1_T_1_←A_3_T_3_ in the case of oligo-^O^G^O^G was found to be fastest. Taking all this into consideration, it can be concluded that the electronic properties of ^OXO^G play a significant role in hole migration through the double helix, in light of its minimal influence on spatial ds-DNA geometry [63].

The electron migration through stacked base pairs in the double helix was found to be different for all the discussed ds-oligonucleotides (as shown in Table 4). As was expected in the case of native oligo-G for all the investigated base pairs dimers, the *k*_HT_ was found at the level of 10^13^–10^15^ (s^−1^). Moreover, as shown in Table 4, the activation energies of A_1_T_1_→G_2_C_2_, G_2_C_2_←A_3_T_3_ and A_3_T_5_←A_5_T_5_ were calculated as below zero eV, which indicates that the mentioned process should be strongly privileged. Comparable results were obtained for oligo-^O^G. However, a slowing down of the electron transfer rate was denoted for A_1_T_1_→G_2_C_2_ by up to 5.70 × 10^9^ (s^−1^). Additionally, the inversion of electron transfer direction A_3_T_5_→A_5_T_5_ in comparison to that of oligo-G (A_3_T_5_←A_5_T_5_) was shown. The appearance of the second ^OXO^G in the ds-oligo (oligo-^O^G^O^G) leads to further *k*_HT_ (s^−1^) decreases of the A_1_T_1_→^OXO^G_2_C_2_ (1.37 × 10^1^) and ^OXO^G_2_C_2_←A_3_T_3_ (5.32 × 10^4^) steps, which indicates that even ^OXO^G_2_ and ^OXO^G_4_ are not equal in terms of electron transfer.

## 4. Discussion

Genetic information is transferred to future generations in the DNA base sequence, but this code of life is continuously exposed to harmful factors whose activity can lead to its damage. Therefore, its stability depends on the effectiveness of repair systems, especially glycosylases, which recognize and remove DNA lesions. According to Barton’s theory, their effectiveness depends on the charge transfer rate between two proteins through π stacked base pairs in the double helix [29], which can be disturbed by DNA lesions such as ^OXO^G.

The privileged role of ^OXO^G as the electron-hole sink has been experimentally shown by Schuster [39,64]. In his work, using a cation radical transfer through ds-DNA induced by anthraquinone, the accumulation of the ^OXO^G oxidation product was noted. Additionally, it was found that the ^OXO^G is the destination point of positive charge migration even though the d[GG]*[CC] base-pair dimer was present in the ds-oligo structure.

Additionally, while the chemistry of isolated lesion removal from DNA is a well-known process, comparatively little is known about its recognition by specific enzymes: how is it possible that 30 copies of MutY (the glycosylase which recognizes an oxoG:::A base pair) can effectively scan an E.coli genome (5 × 10^6^ bp) in less than a few seconds [65]? Barton at al. proposed that single DNA damage localization is performed by proteins through an electron scanning mechanism, i.e., charge transfer (CT) through the DNA double helix [66]. The above has important implications for the efficiency and safety of radiotherapy, as the genome area scanned by a protein is approximately 5300 bases (1800 nm) [67]. However, for many years, charge transfers could only be observed from a distance of up to 200 Å [26].

The above-mentioned observation has been considered theoretically by two independent groups (Voityuk and Siebbeles) [6,7]. An estimate of the electronic properties of all possible trimers was made. Even if this result has yet to be confirmed by systematic experimental studies, it should provide a valuable background for future charge transfer discussion. Additionally, the above-mentioned studies should allow the point of the genome that is susceptible to one-electron oxidizing to be estimated/predicted.

For these reasons a comparative analysis of native ds-oligo (oligo-G) and ds-oligonucleotides containing the isolated (oligo-^O^G) and clustered lesion (oligo-^O^G^O^G) were proposed. The theoretical studies were performed at the M062x/D95**//M062x/6-31++G** level of theory in the aqueous phase (PCM). Non-equilibrated and equilibrated solvent modes were used. For all excitation state calculations, TD-DFT methodology was applied

- In the native oligo-G, the radical cation was located on the 5′-End guanine of the sequence d[A_1_G_2_A_3_G_4_A_5_]*d[T_5_C_4_T_3_C_2_T_1_], which is in good agreement with previous data. Moreover, in the initial vertical cation state (non-equilibrated solvent (NE) mode), the charge and spin were dispersed over both guanines with subsequent rearmament (equilibrated solvent—(EQ) mode) towards the 5′-end one. As expected, the G_2_C_4_ base pair becomes the final point of adiabatic radical formation. Contrary to that, the G_4_C_2_ was exclusively, in all cases, the point of excess electron accumulation. Moreover, the *k*_HT_ of charge transfer through oligo-G showed that electron-hole migration is privileged towards G_2_C_4_, while the extra electron is in a G_4_C_2_ direction. Similar results were obtained from an analysis of energetic barriers.
- ^OXO^G appearing in the double helix structure (oligo-^O^G) confirms previous theoretical and experimental observations. ^OXO^G becomes the sink of the radical cation after one-electron ds-DNA oxidation. Moreover, irrespective of the radical cation’s state, i.e., vertical or adiabatic, and the solvent state—NE or EQ—the positive charge and spin were exclusively located on the ^OXO^G. Additionally, analysis of the energetic barriers and charge rate constant disclosed the preference of electron-hole migration towards the ^OXO^G position in ds-oligo.
- The analysis of clustered DNA damage containing two ^OXO^Gs separated by adenine showed opposite results to those found for the corresponding native oligo-G. The adiabatic ionization potential of this short oligo was found at the same level as that assigned for oligo-^O^G. Firstly, for the initial vertical cation state in the NE and EQ modes, the spin and positive charge were dispersed over three base pairs 5′-^OXO^GA^OXO^G-3′. The system relaxation (adiabatic state) led to spin and charge shift exclusively to the 3′-End ^OXO^G, which is opposite to the results obtained for native oligo, in which spin and charge were located on the 5′-End guanine. Secondly, careful analysis of the adiabatic ionization potential of the base pairs present in oligo-^O^G^O^G showed that 5′-End ^OXO^G_2_ possesses a higher AIP than G_4_ in oligo-G. This indicates that even the discussed oligo containing the symmetric sequence ^OXO^Gs are not equal in terms of electronic properties (^OXO^G_2_ ≠ ^OXO^G_4_).
- A comparative analysis of the positive charge (hole) transfer rate constant (*k*_HT_) revealed a strong relationship to the spatial geometry changes of all the discussed ds-oligos (Table 4). The highest rate constants in (s^−1^) were notably found for A_1_T_1_→G_2_C_2_ and G2C2←A3T3 of oligo-G: 2.2 × 10^12^ and 1.5 × 10^12^, respectively. The presence of ^OXO^dG in the oligo structure slows down the *k*_HT_ as follows: A_3_T_3_→^OXO^G_4_C_4_ (1.0 × 10^9^, 1.26 × 10^9^), ^OXO^G_4_C_4_←A_5_T_5_ (2.3 × 10^8^, 5.15 × 10^7^) of oligo-^O^G and oligo-^O^G^O^G, respectively. The obtained results indicate that ^OXO^G can play a significant role in hole migration through the double helix, in light of its minimal influence on the spatial ds-DNA geometry.
- On the other hand, electron migration through stacked base pairs was found to be different for oligo-G, oligo-^O^G, and oligo-^O^G^O^G (Table 4). The *k*_HT_ was found at a level of 10^13^–10^15^ (s^−1^) for base pair dimers of oligo-G. In the case of oligo-^O^G, a slowing down of up to 5.70 × 10^9^ (s^−1^) of the A_1_T_1_→G_2_C_2_ electron transfer rate was determined for oligo-^O^G. The appearance of the second ^OXO^G in the ds-oligo (oligo-^O^G^O^G) led to further k_HT_ (s^−1^) decreases of the A_1_T_1_→^OXO^G_2_C_2_ (1.37 × 10^1^) and ^OXO^G_2_C_2_←A_3_T_3_ (5.32 × 10^4^) steps, which indicates that even ^OXO^G_2_ and ^OXO^G_4_ are not equal in terms of electron transfer.
- At this point, it is important to mention that even a low dose of radiation at the level of 1 Gy can induce clustered lesions [1,52,53]; hence, it can be expected that tandem lesions such as cdPus can be induced too. CdPus are produced in anaerobic conditions and a hypoxia milieu, which is common for cancer cells, and favors the reaction of carbon-centred or peroxyl nucleotide radicals with the adjacent DNA part [54]. In medical treatment, the most frequent radiodiagnostic techniques use X-rays and gamma radiation, which are classified as Low-LET (Linear Energy Transfer) factors. For example, 131I (a gamma and beta emitter) is commonly used for thyroid therapy and diagnosis [55]. On the other hand, medical centers based on High-LET treatment are becoming of high interest in nuclear medicine–radiotherapy. Following Asaithamby, it can be concluded that High-LET has induced numerous incidents of chromosomal rearrangement [56]. As of 2020, almost 100 such centers exist in 30 countries, with 200,000 patients treated in the USA alone [57]. Therefore, it is important to know how the repair proteins of primary and pathologic/cancer cells recognise the local multiple-damaged sites (LMDS) composed of different kinds of DNA lesions and how LMDS attract repair proteins. For example, a DNA double-strand break (DSB) or an inter- or intrastrand cross-link are repaired by homologous recombination (HR) and nonhomologous end-joining (NHEJ) [58]. The above-mentioned repair mechanisms are already well recognised in the case of isolated lesions, while the recognition and repair process of clustered lesions still requires further advanced studies [59].

Taking all the above into account, it can be postulated that even simple single-stranded clustered DNA damage can significantly change the electronic properties of ds-oligo. Additionally, CDL can influence the charge transfer through the double helix, subsequently disturbing the recognition and repair process of DNA lesions. Because increases in CDL formation have been observed during radio- or chemotherapy, understanding their role in the above processes can be crucial for the efficiency and safety of medical cancer treatment.

## Figures and Tables

**Table 1:** A comparison of differences in spatial geometry between neutral, anionic, and cationic forms of oligo-G, oligo-^O^G, and oligo-^O^G^O^G calculated as the Root Mean Square Deviation (RMSD) of atomic positions (given in (Å^2^)). SP, sugar phosphate.

	System Overlap	RMSD [Å^2^]		
		ds-Oligonucleotides		
		Oligo-G	Oligo-^O^G	Oligo-^O^G^O^G
Anion vs. Neutral	ds-DNA	0.17	0.13	0.11
	Base Pairs	0.16	0.12	0.11
	SP-backbone	0.17	0.15	0.12
Cation vs. Neutral	ds-DNA	0.36	0.31	0.34
	Base Pairs	0.31	0.23	0.29
	SP-backbone	0.42	0.38	0.37

**Table 2:** The electronic parameters in [eV] of oligo-G, oligo-^O^G, and oligo-^O^G^O^G as a complete double helix (**ds-DNA**) or as a base pair stacking system (**BP-system**) calculated at the M062x/6-31++G** level of theory in the aqueous phase. **VIP**—vertical ionization potential, **AIP**—adiabatic ionization potential, **VEAE**—vertical electron attachment energy, **VEA**—vertical electron affinity, **AEA**—adiabatic electron affinity, VEDE—vertical electron detachment energy, **NE**—non-equilibrated, and **EQ**—equilibrated solvent mode. The abbreviations of used parameters’ notation, in detail, have been given in the Materials and Methods part. The row data have been given in Table S1 (Supplementary Materials).

Parameter	ds-Oligonucleotides					
	Oligo-G		Oligo-^O^G		Oligo-^O^G^O^G	
	ds-DNA	BP-System	ds-DNA	BP-System	ds-DNA	BP-System
VIP^NE^	6.72	6.48	6.49	6.27	6.54	6.34
VIP^EQ^	6.08	5.98	5.86	5.79	6.02	5.99
AIP	5.65	5.58	5.39	5.38	5.39	5.40
VEAE^NE^	4.61	4.85	4.40	4.91	−4.40	−4.71
VEAE^EQ^	5.26	5.19	5.04	4.98	−5.04	−4.99
VEA^NE^	−0.84	−0.60	−0.85	−0.60	−0.86	−0.62
VEA^EQ^	−1.58	−1.34	−1.43	−1.35	−1.59	−1.39
AEA	−2.09	−1.90	−2.04	−1.90	−2.09	−1.90
VEDE^NE^	3.36	2.56	3.36	2.62	3.37	2.65
VEDE^EQ^	2.66	2.48	2.67	2.48	2.68	2.49

**Table 3:** Electronic properties in (eV) of isolated base pairs from oligo-G, oligo-^O^G, and oligo-^O^G^O^G, calculated at the M062x/6-31++G** level of theory in the condensed phase (equilibrated solvent mode). The row data have been given in Table S3.

	Oligo-G			Oligo-^O^G			Oligo-^O^G^O^G		
	X_2,4_:G			X_2_:G; X_4_:^OXO^G			X_2,4_:^OXO^G		
	VIP	AIP	VEAE	VIP	AIP	VEAE	VIP	AIP	VEAE
A_1_T_5_	6.65	6.60	−6.60	6.63	6.62	−6.62	6.65	6.63	−6.63
X_2_C_4_	6.13	5.83	−5.51	6.17	6.16	−6.16	5.93	5.93	−5.93
A_3_T_3_	6.65	6.60	−6.60	6.66	6.65	−6.65	6.65	6.65	−6.65
X_4_C_2_	6.13	6.11	−6.11	5.91	5.56	−5.21	5.91	5.56	−5.20
A_5_T_1_	6.74	6.72	−6.73	6.73	6.68	−6.68	6.73	6.68	−6.68
	VEA	AEA	VEDE	VEA	AEA	VEDE	VEA	AEA	VEDE
A_1_T_5_	−1.41	−1.42	1.42	−1.43	−1.43	1.43	−1.42	−1.42	1.43
X_2_C_4_	−1.49	−1.47	1.50	−1.49	−1.50	1.50	−1.51	−1.51	1.52
A_3_T_3_	−1.40	−1.40	1.40	−1.39	−1.39	1.39	−1.39	−1.39	1.39
X_4_C_2_	−1.52	−1.95	2.47	−1.53	−1.97	2.50	−1.53	−1.97	2.50
A_5_T_1_	−1.42	−1.39	1.42	−1.43	−1.40	1.42	−1.43	−1.40	1.43

**Table 4:** The Δ*G*, *λ*, *E_a_*, *V*_12_ (given in eV) and *k*_HT_ (given in s^−1^) of permissible electron hole and excess electron transfer between base pairs, calculated at the M062x/6-31+G* level of theory in the condensed phase. Graphs of “hole” and electron migration through oligo-G, oligo-^O^G, and oligo-^O^G^O^G are also presented in the inserted diagram, with arrows indicating the directions of charge transfer. ^OXO^G is indicated as ^O^G to simplify the notation. The equations of Marcus theory parameters are presented at the beginning of Section 3.6. The row data have been given in Table S5a band Table S5b.

Electron Transfer											
	Oligo-G					Oligo-^O^G					
System	*λ*	Δ*G*	*E_a_*	*V*_12_	*k*_HT_	System	*λ*	Δ*G*	*E_a_*	*V*_12_	*k*_HT_
A_1_T_1_→G_2_C_2_	−0.02	−0.05	−0.06	0.02	0.00	A_1_T_1_→G_2_C_2_	0.01	−0.07	0.21	0.01	5.70 × 10^9^
G_2_C_2_←A_3_T_3_	−0.02	−0.07	−0.11	0.04	0.00	G_2_C_2_←A_3_T_3_	0.00	−0.10	−8.25	0.05	0.00
A_3_T_3_→G_4_C_4_	0.43	−0.54	0.01	0.07	8.75 × 10^13^	A_3_T_3_→^O^G_4_C_4_	0.43	−0.57	0.01	0.06	6.40 × 10^13^
G_4_C_4_←A_5_T_5_	0.46	−0.55	0.01	0.04	4.04 × 10^13^	^O^G_4_C_4_←A_5_T_5_	0.45	−0.56	0.01	0.04	3.05 × 10^13^
A_1_T_1_←A_3_T_3_	0.01	−0.02	0.004	0.09	1.2 × 10^15^	A_1_T_1_←A_3_T_3_	3 × 10^−3^	−0.03	0.09	0.09	6.9 × 10^13^
G_2_C_2_→G_4_C_4_	0.46	−0.47	0.00	0.05	4.9 × 10^13^	G_2_C_2_→^O^G_4_C_4_	0.43	−0.47	0.001	0.05	6.2 × 10^13^
A_3_T_5_←A_5_T_5_	−0.02	−0.01	−0.01	0.08	0.00	A_3_T_5_→A_5_T_5_	−0.03	−0.01	−0.01	0.08	0.00
	**oligo−^O^G^O^G**					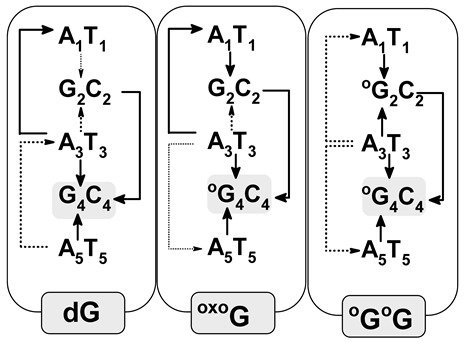					
A_1_T_1_→^O^G_2_C_2_	2.2 × 10^−3^	−0.08	0.75	0.01	1.37 × 10^1^						
^O^G_2_C_2_←A_3_T_3_	0.01	−0.12	0.60	0.06	5.32 × 10^4^						
A_3_T_3_→^O^G_4_C_4_	0.44	−0.58	0.01	0.07	7.41 × 10^13^						
^O^G_4_C_4_←A_5_T_5_	0.45	−0.56	0.01	0.05	4.28 × 10^13^						
A_1_T_1_←A_3_T_3_	−2.5 × 10^−3^	−0.04	−0.17	0.02	0.00						
G_2_C_2_→^O^G_4_C_4_	0.44	−0.46	2.0 × 10^−4^	0.05	6.87 × 10^13^						
A_3_T_5_→A_5_T_5_	−0.02	−0.02	−0.02	0.07	0.00						
**Hole Transfer**											
	**oligo-G**					**oligo-^O^G**					
**System**	***λ***	**Δ*G***	***E_a_***	***V*** **_12_**	***k*_HT_**	**System**	***λ***	**Δ*G***	***E_a_***	***V*** **_12_**	***k*_HT_**
A_1_T_1_→G_2_C_2_	0.30	−0.77	0.18	0.30	2.2 × 10^12^	A_1_T_1_→G_2_C_2_	0.00	−0.46	11.08	0.29	0.00
G_2_C_2_←A_3_T_3_	0.30	−0.77	0.18	0.24	1.5 × 10^12^	G_2_C_2_←A_3_T_3_	0.01	−0.49	8.29	0.23	0.00
A_3_T_3_→G_4_C_4_	0.03	−0.49	2.05	0.28	0.00	A_3_T_3_→^O^G_4_C_4_	0.35	−1.09	0.40	0.41	1.0 × 10^9^
G_4_C_4_←A_5_T_5_	0.01	−0.61	7.69	0.29	0.00	^O^G_4_C_4_←A_5_T_5_	0.34	−1.12	0.44	0.35	2.3 × 10^8^
A_1_T_1_←A_3_T_3_	0.05	−0.00	0.01	0.02	2.1 × 10^11^	A_1_T_1_←A_3_T_3_	0.01	−0.03	0.01	0.01	1.4 × 10^13^
G_2_C_2_←G_4_C_4_	0.29	−0.28	2.0 × 10^−4^	0.01	3.6 × 10^10^	G_2_C_2_→^O^G_4_C_4_	0.35	−0.60	0.05	0.12	7.5 × 10^13^
A_3_T_3_←A_5_T_5_	0.05	−0.12	0.02	0.04	6.2 × 10^13^	A_3_T_3_←A_5_T_5_	0.01	−0.02	0.003	0.04	1.9 × 10^14^
	**oligo-^O^G^O^G**					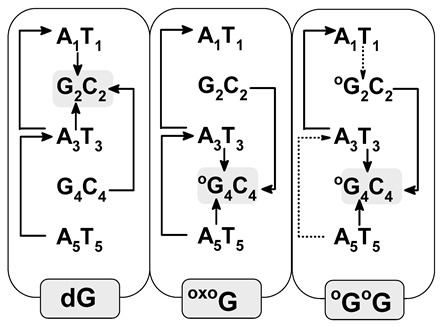					
A_1_T_1_→^O^G_2_C_2_	−5.0 × 10^−5^	−0.69	−2.2 × 10^3^	0.39	0.00						
^O^G_2_C_2_←A_3_T_3_	1.7 × 10^−4^	−0.72	76.06	0.35	0.00						
A_3_T_3_→^O^G _4_C_4_	0.35	−1.09	0.39	0.41	1.26 × 10^9^						
^O^G_4_C_4_←A_5_T_5_	0.35	−1.12	0.42	0.16	5.15 × 10^7^						
A_1_T_1_←A_3_T_3_	0.02	−0.03	2.0 × 10^−4^	0.30	1.03 × 10^16^						
^O^G_2_C→^O^G _4_C_4_	0.35	−0.38	3.6 × 10^−4^	0.01	4.07 × 10^12^						
A_3_T_5_→A_5_T_5_	−0.02	−0.017	−0.02	0.07	0.00						

## Supplementary Materials

The following supporting information can be downloaded at: https://www.mdpi.com/article/10.3390/biom13030517/s1, Table S1: The energies (in Hartree) of neural, vertical cation (VC^NC^) (NE-non-equilibrated), vertical cation (VC^EQ^) (EQ-equilibrated), vertical anion (VA^NE^), vertical anion (VA^EQ^), adiabatic cation (AC), adiabatic anion (AA) and vertical neutral from cation (VNC^NE^), vertical neutral from cation (VNC^EQ^), vertical neutral from anion (VNA^NE^), vertical neutral from anion (VNA^EQ^) of complete DNA double helix and base pairs skeleton extracted from *ds*-oligonucleotides calculated at the M06-2x/6-31+G** and M06-2x/6-31++G** level of theory in the aqueous phase, respectively. Table S2: Hirshfeld charge and spin distribution in the shape of ds-oligonucleotides, only nucleosides bases were taken into consideration, calculated at the M06-2x/6-31++G** level of theory in the aqueous phase. Vertical cation (VC^NC^) (NE-non-equilibrated), vertical cation (VC^EQ^) (EQ-equilibrated), vertical anion (VA^NE^), vertical anion (VA^EQ^), adiabatic cation (AC), adiabatic anion (AA) and vertical neutral from cation (VNC^NE^), vertical neutral from cation (VNC^EQ^), vertical neutral from anion (VNA^NE^), vertical neutral from anion (VNA^EQ^). Table S3: The energies (in Hartree) of neural, vertical cation, adiabatic cation, and vertical neutral forms of base pairs extracted from ds-oligonucleotides calculated at the M06-2x/6-31++G** level of theory in the aqueous phase. Table S4: The energy barriers (in eV) for radical cation migration between base pairs within trimers. Vertical (Vert) mode, i.e., the energies of each base pair’s radical cation, which were calculated for their neutral geometry. Adiabatic (Adia) mode, i.e., the energies of each base pair’s radical cation were calculated for their cation geometry. Arrows indicate the direction of electron hole or excess electron transfer from one base pair to another, e.g., A^+^→G calculated at M06-2x/6-31++G** level of theory in the aqueous phase. Table S5a: The energies: ground (E^GR^) and excitation (E^EX^) state energies and excitation and HOMO energies as well as corresponding dipole moments ground, excitation, and transition (DM^G^, DM^EX^, D_12_) in Debays of neighbor base pair extracted from selected dimmers of *ds*-oligonucleotides, calculated at the M06-2x/6-31++G** level of theory in the aqueous phase using the DFT or TD-DFT methodology. Table S5b: The energies: ground (E^GR^) and excitation (E^EX^) state energies and excitation and HOMO energies as well as corresponding dipole moments ground, excitation, and transition (DM^G^, DM^EX^, D_12_) in Debays of distal base pair extracted from selected trimers of *ds*-oligonucleotides, calculated at the M06-2x/6-31++G** level of theory in the aqueous phase using the DFT or TD-DFT methodology. The cartesian coordinates of discussed oligo structure have been attached as follows: oligo-G: (1) oligo-G_Neutral, (2) oligo-G_Cation, (3) oligo-G_Anion, oligo-^O^G: (4) oligo-oG_Neutral, (5) oligo-oG_Cation, (6) oligo-oG_Anion, oligo-^O^G^O^G: (7) oligo-oGoG_Neutral, (8) oligo-oGoG_Cation, (9) oligo-oGoG_Anion.
