# Supplementary material for: How Clustered DNA Damage Can Change the Electronic Properties of ds-DNA—Differences between GAG, GAOXOG, and OXOGAOXOG
Source: Biomolecules. 2023 Mar 11;13(3):517. doi: 10.3390/biom13030517 (PMC10046028; doi:10.3390/biom13030517)
Supplement: Supplementary file 1 [file biomolecules-13-00517-s001.zip › biomolecules-2215358-supplementary.pdf]

Table S1. The energies (in Hartree) of Neural, Vertical Cation (VC^{NC}) (NE-non-equilibrated), Vertical Cation (VC^{EQ}) (EQ-equilibrated), Vertical Anion (VA^{NE}), Vertical Anion (VA^{EQ}), Adiabatic Cation (AC), Adiabatic Anion (AA) and Vertical Neutral from Cation (VNC^{NE}), Vertical Neutral from Cation (VNC^{EQ}), Vertical Neutral from Anion (VNA^{NE}), Vertical Neutral from Anion (VNA^{EQ}) of complete DNA double helix and base pairs skeleton extracted from *ds*-oligonucleotides calculated at the M06-2x/6-31+G** and M06-2x/6-31++G** level of theory in the aqueous phase, respectively.

	**Neutrał**	**VC^{NE}**	**VC^{EQ}**	**VA^{NE}**	**VA^{EQ}**	**AC**	**AA**	**VNC^{NE}**	**VNC^{EQ}**	**VNA^{NE}**	**VNA^{EQ}**
	**Complete DNA double helix**										
**oligo-G**	-12776,3427	-12776,10	-12776,1	-12776,37	-12776,40072	-12776,135	-12776,41959	-12776,30422	-12776,32834	-12776,29604	-12776,32187
**oligo-^{O}G**	-12851,5718	-12851,33	-12851,4	-12851,60	-12851,62442	-12851,374	-12851,64682	-12851,5356	-12851,55903	-12851,52334	-12851,54879
**oligo-^{O}G^{O}G**	-12926,7992	-12926,56	-12926,6	-12926,83	-12926,8577	-12926,601	-12926,8759	-12926,7627	-12926,78621	-12926,75207	-12926,77740
**Base Pairs skeleton extracted from *ds*-oligonucleotides**											
**oligo-G**	-4638,177641	-4637,94	-4637,96	-4638,2	-4638,226832	-4637,9727	-4638,247303	-4638,15103	-4638,163199	-4638,15	-4638,16
**oligo-^{O}G**	-4713,405801	-4713,18	-4713,19	-4713,428	-4713,455462	-4713,2082	-4713,475657	-4713,388742	-4713,39119	-4713,379418	-4713,38
**oligo-^{O}G^{O}G**	-4788,633204	-4788,40	-4788,41	-4788,656	-4788,684321	-4788,4349	-4788,703127	-4788,607882	-4788,618409	-4788,60561	-4788,61

Table S2. Hirshfeld charge and spin distribution in the shape of *ds*-oligonucleotides, only nucleosides bases were taken into consideration, calculated at the M06-2x/6-31++G** level of theory in the aqueous phase. Vertical Cation (VC^{NC}) (NE-non-equilibrated), Vertical Cation (VC^{EQ}) (EQ-equilibrated), Vertical Anion (VA^{NE}), Vertical Anion (VA^{EQ}), Adiabatic Cation (AC), Adiabatic Anion (AA) and Vertical Neutral from Cation (VNC^{NE}), Vertical Neutral from Cation (VNC^{EQ}), Vertical Neutral from Anion (VNA^{NE}), Vertical Neutral from Anion (VNA^{EQ})

**oligo-G**									
	**Neutal**	**VC^{NE}**		**VC^{EQ}**		**AC**		**VNC^{NE}**	**NVC^{EQ}**
	**Charge**	**Charge**	**Spin**	**Charge**	**Spin**	**Charge**	**Spin**	**Charge**	**Charge**
**A_1T_5**	-0.03	0.03	0.03	0.08	0.07	0.10	0.06	-0.02	-0.01
**G_2C_4**	0.03	0.39	0.39	0.85	0.90	0.84	0.92	0.04	0.01
**A_3T_3**	-0.03	0.08	0.09	0.02	0.02	0.03	0.02	-0.02	-0.03
**G_4C_2**	0.04	0.47	0.48	0.07	0.02	0.05	0.00	0.03	0.04
**A_5T_1**	-0.01	0.03	0.01	-0.01	0.00	-0.01	0.00	-0.03	-0.01
**oligo-^{O}G**									
**A_1T_5**	-0.02	0.00	0.00	-0.01	0.00	-0.01	0.00	0.00	-0.01
**G_2C_4**	0.02	0.04	0.00	0.04	0.00	0.04	0.00	0.03	0.03
**A_3T_3**	-0.02	0.08	0.09	0.07	0.06	0.07	0.05	-0.01	-0.01
**$^{oxo}G_4C_2$**	0.02	0.80	0.89	0.85	0.91	0.83	0.93	-0.02	0.00
**A_5T_1**	0.00	0.07	0.02	0.06	0.02	0.06	0.02	0.00	-0.01
**oligo-^{O}G^{O}G**									
**A_1T_5**	-0.01	0.07	0.03	0.01	0.03	0.01	0.00	-0.01	0.00
**$^{oxo}G_2C_4$**	0.000	0.43	0.48	0.43	0.47	0.13	0.10	0.01	0.01
**A_3T_3**	-0.01	0.10	0.09	0.10	0.09	0.08	0.05	0.01	0.00
**$^{oxo}G_4C_2$**	0.03	0.38	0.39	0.41	0.41	0.73	0.83	0.01	0.00
**A_5T_1**	0.00	0.04	0.01	0.03	0.01	0.06	0.02	-0.02	-0.01
**oligo-G**									
	**VA^{NE}**		**VA^{EQ}**		**AA**		**VNA^{NE}**	**NVA^{EQ}**	
	Charge	Spin	Charge	Spin	Charge	Spin	Charge	Charge	
**A_1T_5**	-0.05	0.00	-0.04	0.00	-0.02	0.00	-0.01	-0.02	
**G_2C_4**	0.01	0.01	0.01	0.02	0.01	0.00	0.02	0.01	
**A_3T_3**	-0.11	0.06	-0.16	0.11	-0.09	0.02	-0.05	-0.04	
**G_4C_2**	-0.70	0.83	-0.61	0.70	-0.82	0.94	0.02	0.05	
**A_5T_1**	-0.15	0.10	-0.21	0.17	-0.08	0.04	0.01	-0.01	
**oligo-^{O}G**									
**A_1T_5**	-0.04	0.00	-0.02	0.00	-0.02	0.00	-0.01	-0.02	
**G_2C_4**	-0.01	0.02	0.00	0.02	0.01	0.00	0.02	0.01	
**A_3T_3**	-0.11	0.07	-0.15	0.11	-0.09	0.02	-0.04	-0.04	
**$^{oxo}G_4C_2$**	-0.71	0.81	-0.66	0.74	-0.82	0.94	0.02	0.04	
**A_5T_1**	-0.14	0.09	-0.17	0.14	-0.07	0.03	0.02	0.00	
**oligo-^{O}G^{O}G**									
**A_1T_5**	-0.09	0.06	-0.01	0.00	-0.01	0.00	-0.00	-0.01	
**$^{oxo}G_2C_4$**	-0.22	0.22	-0.02	0.01	-0.02	0.00	0.00	-0.01	
**A_3T_3**	-0.15	0.13	-0.11	0.08	-0.08	0.02	-0.03	-0.03	
**$^{oxo}G_4C_2$**	-0.45	0.52	-0.70	0.79	-0.81	0.94	0.02	0.05	
**A_5T_1**	-0.10	0.06	-0.16	0.12	-0.07	0.03	0.02	0.01	

Table S3. The energies (in Hartree) of Neural, Vertical Cation, Adiabatic Cation and Vertical Neutral forms of base pairs extracted from *ds*-oligonucleotides calculated at the M06-2x/6-31++G** level of theory in the aqueous phase.

	Neutral	Vertical Cation	Adiabatic Cation	Vert Neutral
**oligo-G**				
**A_1T_5**	-921,191682	-920,947308	-920,949073	-921,19147
**G_2C_4**	-937,252595	-937,027434	-937,038223	-937,240567
**A_3T_3**	-921,192489	-920,948177	-920,949811	-921,192176
**G_4C_2**	-937,252759	-937,027507	-937,028165	-937,252764
**A_5T_1**	-921,191961	-920,944427	-920,945028	-921,192194
**oligo-G**		**Vertical Anion**	**Adiabatic Anion**	**Vert Neutral**
**A_1T_5**		-921,243655	-921,243963	-921,191767
**G_2C_4**		-937,307348	-937,306719	-937,25153
**A_3T_3**		-921,243775	-921,24406	-921,192473
**G_4C_2**		-937,308484	-937,324241	-937,233582
**A_5T_1**		-921,244136	-921,243109	-921,190968
**oligo-^{O}G**	**Neutral**	**Vertical Cation**	**Adiabatic Cation**	**Vert Neutral**
**A_1T_5**	-921,191848	-920,948067	-920,94861	-921,191884
**G_2C_4**	-937,251538	-937,024955	-937,025162	-937,251507
**A_3T_3**	-921,19218	-920,94745	-920,94771	-921,192126
**G_4C_2**	-1012,478383	-1012,261293	-1012,274085	-1012,465472
**A_5T_1**	-921,192039	-920,944573	-920,946723	-921,19222
**oligo-^{O}G**		**Vertical Anion**	**Anion**	**Vert Neutral**
**A_1T_5**		-921,244334	-921,244284	-921,191793
**G_2C_4**		-937,306431	-937,306562	-937,251406
**A_3T_3**		-921,243124	-921,243402	-921,192323
**G_4C_2**		-1012,53476	-1012,550605	-1012,458617
**A_5T_1**		-921,244425	-921,24351	-921,191203
**oligo-^{O}G^{O}G**	**Neutral**	**Vertical Cation**	**Adiabatic Cation**	**Vert Neutral**
**A_1T_5**	-921,191698	-920,947423	-920,948228	-921,191759
**G_2C_4**	-1012,476983	-1012,258891	-1012,25895	-1012,476979
**A_3T_3**	-921,192189	-920,947935	-920,947765	-921,192186
**G_4C_2**	-1012,47839	-1012,261184	-1012,274135	-1012,465399
**A_5T_1**	-921,19209	-920,944778	-920,946598	-921,192154
**oligo-^{O}G^{O}G**		**Vertical Anion**	**Anion**	**Vert Neutral**
**A_1T_5**		-921,243959	-921,244043	-921,19165
**G_2C_4**		-1012,532389	-1012,532423	-1012,476736
**A_3T_3**		-921,243165	-921,243122	-921,192013
**G_4C_2**		-1012,53476	-1012,550638	-1012,458849
**A_5T_1**		-921,244562	-921,243653	-921,191277

Table S4. The energy barriers (in eV) for radical cation migration between base pairs within trimers. Vertical (**Vert**) mode, i.e. the energies of each base pair's radical cation, which were calculated for their neutral geometry. Adiabatic (**Adia**) mode i.e. the energies of each base pair's radical cation were calculated for their cation geometry. Arrows indicate direction of Electron-hole or Excess Electron Transfer from one base pair to another e.g., $A^+ \rightarrow G$ calculated at M06-2x/6-31++G** level of theory in the aqueous phase.

				**Electron-hole transfer**					
		$A_1 \leftarrow G_2$	$A_1 \rightarrow G_2$	$G_2 \leftarrow A_3$	$G_2 \rightarrow A_3$	$A_3 \leftarrow G_4$	$A_3 \rightarrow G_4$	$G_4 \leftarrow A_5$	$G_4 \rightarrow A_5$
**oligo-G**	Vert	1,14	-0,47	-0,47	1,14	0,54	-0,47	-0,60	0,62
	Adia	0,77	-0,77	-0,77	0,77	0,49	-0,49	-0,61	0,61
		$A_1 \leftarrow G_2$	$A_1 \rightarrow {}^{O}G_2$	${}^{O}G_2 \leftarrow A_3$	${}^{O}G_2 \rightarrow A_3$	$A_3 \leftarrow G_4$	$A_3 \rightarrow G_4$	$G_4 \leftarrow A_5$	$G_4 \rightarrow A_5$
**oligo-^{O}G**	Vert	0,47	-0,45	-0,49	0,50	1,45	-0,74	-0,77	1,53
	Adia	0,46	-0,46	-0,49	0,49	1,09	-1,09	-1,09	1,12
		$A_1 \leftarrow G_2$	$A_1 \rightarrow {}^{O}G_2$	${}^{O}G_2 \leftarrow A_3$	${}^{O}G_2 \rightarrow A_3$	$A_3 \leftarrow {}^{O}G_4$	$A_3 \rightarrow {}^{O}G_4$	${}^{O}G_4 \leftarrow A_5$	${}^{O}G_4 \rightarrow A_5$
**oligo-^{O}G^{O}G**	Vert	0,71	-0,69	-0,72	0,71	1,44	-0,74	-0,77	1,53
	Adia	0,69	-0,69	-0,72	0,72	1,09	-1,09	-1,12	1,12
		$A_1 \leftarrow A_3$	$A_1 \rightarrow A_3$	$G_2 \leftarrow G_4$	$G_2 \rightarrow G_4$	$A_3 \leftarrow A_5$	$A_3 \rightarrow A_5$		
**oligo-G**	Vert	0,05	0,05	0,02	0,30	-0,07	0,04		
	Adia	0,00	0,00	-0,28	0,28	-0,12	0,12		
		$A_1 \leftarrow A_3$	$A_1 \rightarrow A_3$	${}^{O}G_2 \leftarrow G_4$	${}^{O}G_2 \rightarrow G_4$	$A_3 \leftarrow A_5$	$A_3 \rightarrow A_5$		
**oligo-^{O}G**	Vert	-0,02	0,01	0,61	-0,25	-0,02	0,01		
	Adia	-0,03	0,03	0,60	-0,60	-0,02	0,02		
		$A_1 \leftarrow A_3$	$A_1 \rightarrow A_3$	${}^{O}G_2 \leftarrow {}^{O}G_4$	${}^{O}G_2 \rightarrow {}^{O}G_4$	$A_3 \leftarrow A_5$	$A_3 \rightarrow A_5$		
**oligo-^{O}G^{O}G**	Vert	0,00	0,02	0,38	-0,02	0,32	0,43		
	Adia	0,03	0,03	0,37	-0,37	-0,03	0,03		
				**Excess electron transfer**					
		$A_1 \leftarrow G_2$	$A_1 \rightarrow G_2$	$G_2 \leftarrow A_3$	$G_2 \rightarrow A_3$	$A_3 \leftarrow G_4$	$A_3 \rightarrow G_4$	$G_4 \leftarrow A_5$	$G_4 \rightarrow A_5$
**oligo-G**	Vert	0,09	-0,07	-0,09	0,11	1,07	-0,11	-0,10	1,05
	Adia	0,05	-0,05	-0,07	0,07	0,54	-0,54	-0,55	0,55
		$A_1 \leftarrow G_2$	$A_1 \rightarrow {}^{O}G_2$	${}^{O}G_2 \leftarrow A_3$	${}^{O}G_2 \rightarrow A_3$	$A_3 \leftarrow G_4$	$A_3 \rightarrow G_4$	$G_4 \leftarrow A_5$	$G_4 \rightarrow A_5$
**oligo-^{O}G**	Vert	0,07	-0,07	-0,10	0,11	1,12	-0,14	-0,11	1,08
	Adia	0,07	-0,07	-0,10	0,10	0,57	-0,57	-0,56	0,56
		$A_1 \leftarrow G_2$	$A_1 \rightarrow {}^{O}G_2$	${}^{O}G_2 \leftarrow A_3$	${}^{O}G_2 \rightarrow A_3$	$A_3 \leftarrow {}^{O}G_4$	$A_3 \rightarrow {}^{O}G_4$	${}^{O}G_4 \leftarrow A_5$	${}^{O}G_4 \rightarrow A_5$
**oligo-^{O}G^{O}G**	Vert	0,08	-0,08	-0,12	0,13	1,11	-0,14	-0,11	1,07
	Adia	0,09	-0,08	-0,12	0,12	0,58	-0,58	-0,56	0,56
		$A_1 \leftarrow A_3$	$A_1 \rightarrow A_3$	$G_2 \leftarrow G_4$	$G_2 \rightarrow G_4$	$A_3 \leftarrow A_5$	$A_3 \rightarrow A_5$		
**oligo-G**	Vert	-0.01	0.03	0.46	-0.04	0.00	0.01		
	Adia	-0.02	0.02	0.47	-0.47	-0.01	0.01		
		$A_1 \leftarrow A_3$	$A_1 \rightarrow A_3$	${}^{O}G_2 \leftarrow G_4$	${}^{O}G_2 \rightarrow G_4$	$A_3 \leftarrow A_5$	$A_3 \rightarrow A_5$		
**oligo-^{O}G**	Vert	-0.03	0.00	0.47	-0.04	0.01	0.01		
	Adia	-0.03	0.03	0.47	-0.47	0.01	-0.01		
		$A_1 \leftarrow A_3$	$A_1 \rightarrow A_3$	${}^{O}G_2 \leftarrow {}^{O}G_4$	${}^{O}G_2 \rightarrow {}^{O}G_4$	$A_3 \leftarrow A_5$	$A_3 \rightarrow A_5$		
**oligo-^{O}G^{O}G**	Vert	-0,04	0,04	0,46	-0,03	0,02	0,00		
	Adia	-0,04	0,04	0,46	-0,46	0,02	-0,02		

Table S5a. The Energies: Ground (E^{GR}) and Excitation (E^{EX}) state energies and Excitation and HOMO Energies as well as corresponding Dipole Moments Ground, Excitation and Transition (DM^{G}, DM^{EX}, D_{12}) in Debays of neighbour base pair extracted from selected dimmers of *ds*-oligonucleotides, calculated at the M06-2x/6-31++G** level of theory in the aqueous phase using the DFT or TD-DFT methodology.

SYSTEM	Base Pair Dimer	E^{GR}	DM^{GR}	E^{EX}	DM^{EX}	Sing.S.E^{EX}	E^{HOMO}	E^{HOMO-1}	E^{LUMO}	E^{LUMO-1}
oligo-G	$A_1 \Vert G_2$	-1858,467934	9,97	-1858,336047	10,31	4,92	-0,2606	-0,2786	-0,0162	-0,0129
	$G_2 \Vert A_3$	-1858,468344	9,29	-1858,334475	9,33	5,00	-0,2606	-0,2786	-0,0162	-0,0129
	$A_3 \Vert G_4$	-1858,467944	10,09	-1858,334834	10,45	5,01	-0,2590	-0,2799	-0,0164	-0,0115
	$G_4 \Vert A_5$	-1858,468728	9,94	-1858,335837	9,92	5,06	-0,2612	-0,2824	-0,0170	-0,0138
oligo-^{O}G	$A_1 \Vert G_2$	-1858,468443	10,06	-1858,336412	10,42	4,92	-0,2591	-0,2807	-0,0156	-0,0149
	$G_2 \Vert A_3$	-1858,466571	9,09	-1858,332663	9,12	4,99	-0,2619	-0,2786	-0,0166	-0,0129
	$A_3 \Vert {}^{oxo}G_4$	-1933,694205	16,24	-1933,568074	14,53	4,81	-0,2519	-0,2831	-0,0169	-0,0121
	${}^{oxo}G_4 \Vert A_5$	-1933,695763	16,45	-1858,335837	9,92	4,90	-0,2532	-0,2836	-0,0176	-0,0142
oligo-^{O}G^{O}G	$A_1 \Vert G_2$	-1933,694886	16,27	-1933,568844	14,53	4,43	-0,2528	-0,2830	-0,0162	-0,0151
	${}^{oxo}G_2 \Vert A_3$	-1933,692729	15,73	-1933,567003	14,06	4,94	-0,2537	-0,2796	-0,0175	-0,0134
	$A_3 \Vert {}^{oxo}G_4$	-1933,694324	16,24	-1933,568137	14,53	4,83	-0,2523	-0,2827	-0,0170	-0,0120
	${}^{oxo}G_4 \Vert A_5$	-1933,695805	16,45	-1933,569079	14,77	4,90	-0,2534	-0,2835	-0,0177	-0,0142

Table S5b. The Energies: Ground (E^{GR}) and Excitation (E^{EX}) state energies and Excitation and HOMO Energies as well as corresponding Dipole Moments Ground, Excitation and Transition (DM^{G}, DM^{EX}, D_{12}) in Debays of distal base pair extracted from selected trimmers of *ds*-oligonucleotides, calculated at the M06-2x/6-31++G** level of theory in the aqueous phase using the DFT or TD-DFT methodology

SYSTEM	Base Pair Dimer	E^{GR}	DM^{GR}	E^{EX}	DM^{EX}	D_{12}	E^{HOMO}	E^{HOMO-1}	E^{LUMO}	E^{LUMO-1}
oligo-G	$A_1 \Vert A_3$	-1858,467934	9,97	-1858,336047	10,31	6,40	-0,25828	-0,2805	-0,0151	-0,01392
	$G_2 \Vert G_4$	-1858,468344	9,29	-1858,334475	9,33	11,39	-0,26062	-0,27856	-0,01622	-0,01292
	$A_3 \Vert A_5$	-1858,467944	10,09	-1858,334834	10,45	11,13	-0,25900	-0,2799	-0,01643	-0,01148
oligo-^{O}G	$A_1 \Vert A_3$	-1858,468443	10,06	-1858,336412	10,42	6,17	-0,25909	-0,28071	-0,01555	-0,01486
	$G_2 \Vert {}^{oxo}G_4$	-1858,466571	9,09	-1858,332663	9,12	11,64	-0,26195	-0,27864	-0,01665	-0,01294
	$A_3 \Vert A_5$	-1933,694205	16,24	-1933,568074	14,53	3,59	-0,25192	-0,28312	-0,01686	-0,01208
oligo-^{O}G^{O}G	$A_1 \Vert A_3$	-1842,384666	3,49	-1842,250666	3,93	9,71	-0,25828	-0,2805	-0,0151	-0,01392
	${}^{oxo}G_4 \Vert {}^{oxo}G_4$	-2024,956101	24,37	-2024,82773	22,40	7,12	-0,25658	-0,25748	-0,01946	-0,01558
	$A_3 \Vert A_5$	-1842,385053	3,60	-1842,251528	3,72	10,06	-0,28370	-0,28676	-0,01831	-0,01281
